# It Do Move

**Published:** 1895-12

**Authors:** 


"IT DO MOVE."—The London *Lancet*, anent Homœopathy, and in answer to a correspondent, says:

"The homœopaths isolate themselves by adopting an exclusive theory of medicine, which, after a hundred years and in the broadest age of medicine, is as far off recognition as it ever was."

About four hundred years ago, more or less, a certain gentleman announced that the earth revolved around the sun. The high priests of orthodoxy were scandalized at this "exclusive" and heretical theory. And that theory "is as far off recognition to-day as it ever was" in the mind of the Rev. John Jasper, African minister of the gospel, who insists of the sun that "it do move." This is undoubtedly "the broadest age of medicine" that ever was; it is so broad that it embraces in its fold every notion conceived in the medical brain—from "serum" to blood-letting—but it cannot take in the truth. May it not be possible that the "broadness" on which men so plume themselves is, after all, something not worth bragging about? Something that no one need be proud of? Is not the true way *in everything* straight and rather narrow? A man's mind may be so broad as to take in hundreds of solutions to the problem, 2 x 2=?, but for all that there is but one correct solution. Truth is of necessity exclusive, excluding everything that is in conflict with itself.—*Homœopathic Envoy.*

